# Maintenance of cytomegalovirus-specific CD4^pos ^T-cell response in rheumatoid arthritis patients receiving anti-tumor necrosis factor treatments

**DOI:** 10.1186/ar3083

**Published:** 2010-07-15

**Authors:** Jean-Luc Davignon, Jean-Frédéric Boyer, Bénédicte Jamard, Delphine Nigon, Arnaud Constantin, Alain Cantagrel

**Affiliations:** 1 JE 2510, University Paul Sabatier Toulouse III. France, IFR 150, CPTP, Bâtiment C, CHU Purpan, 1, place Baylac, 31300 Toulouse, France; 2 Centre of Rheumatology, CHU Purpan, 1, place Baylac, BP 3028, 31024 Toulouse, France; 3 INSERM U558, Toulouse, Faculté de Médecine, 37, allées Jules Guesde, 31073 Toulouse Cedex, France

## Abstract

**Introduction:**

Anti-tumor necrosis factor (TNF)-α biotherapies have considerably changed the treatment of rheumatoid arthritis (RA). However, serious infections are a major concern in patients with rheumatic diseases treated with anti-TNF-α. Little is known about viral, especially latent, infections in anti-TNF-α treatments. Infections by cytomegalovirus (CMV), a β-herpes virus, are frequent and induce a strong CD4^pos ^T-cell immunity, which participates in the control of infection. We thus have chosen to analyze the CD4^pos ^T-cell response to CMV antigens as a model of antiviral response in RA patients treated with anti-TNF-α. CD28 expression was evaluated.

**Methods:**

We have measured the CD4^pos ^response to CMV antigens in RA patients, before and after initiation of treatment with an anti-TNF-α agent. The intracellular production of interferon (IFN)-γ in total and CD28^neg ^CD4^pos ^T cells in response to CMV antigens (Ags) was evaluated with flow cytometry. The proliferation of total CD4^pos ^T cells in the presence of CMV antigens was measured with ^3^H-thymidine incorporation.

**Results:**

Anti-TNF-α treatments impaired neither the anti-CD4^pos ^anti-CMV IFN-γ response nor the proliferative response in patients. The percentage of CD28^neg ^CD4^pos ^cells remained constant.

**Conclusions:**

Our data suggest that the CD4^pos ^T-cell response against CMV is not altered by anti-TNF-α treatments and that infection remains controlled in treated RA patients latently infected with CMV. Our observation brings new insight into the current knowledge of the risks of infection in patients treated with anti-TNF-α biotherapies.

## Introduction

Current therapies for RA are aimed at inhibiting inflammatory cytokines, especially tumor necrosis factor (TNF)-α biotherapies, such as antibodies (infliximab, adalimumab) and soluble receptor (etanercept) specific for TNF. These three commercially available TNF antagonists have been tested in established and in early diseases. They effectively improved disease activity and significantly slowed radiologic deterioration [1,2]. However, serious infections are a major concern in patients with rheumatic diseases, and inhibition of TNF-α increases the risk of serious and benign infections [3]. The role played by TNF-α in the body's defense against bacterial and viral invasion is multiple: recruitment of neutrophils, eosinophils, and macrophages; release of cytokines and local chemokines; attraction and activation of phagocytes; increased T-cell adhesion; enhanced antigen presentation; and recruitment and proliferation of T and B cells [4]. Moreover, TNF-α is also involved in the formation and sustainment of Mycobacterium granulomatous lesions [5]. Neutralization of TNF-α for treating rheumatic diseases increases the risk of reactivation and outbreak of tuberculosis and other opportunistic infections [6,7]. A decrease of the tuberculosis-specific CD4^pos ^T-cell response in patients treated with anti-TNF was found [8]. In addition, anti-TNF treatment induced a reduction in effector memory CD8^pos ^T cells specific for mycobacteria [9].

In contrast, less is known about viral infections. Herpesviruses can persist in patients in a latent state and be reactivated under situations of immunosuppression. Although cases of lymphoproliferative disorders have been reported in RA, the role of TNF-α antagonists in Epstein-Barr virus (EBV)-related lymphomas is not clear [3,10,11]. Conversely, inflammation, a hallmark of RA, might be associated with the risk of lymphoma [12]. Regarding EBV infections, data are rather reassuring. In a recent study, no impairment of the anti-EBV CD8^pos ^T-cell response was found in patients treated with anti-TNF, and the EBV viral load was not increased [13]. However, regarding varicella zoster virus, another herpesvirus, a recent publication suggested that anti-TNF-α antibodies could be associated with increased risk of reactivation, responsible for an increased rate of herpes zoster events in patients treated with these biologic agents [14].

Cytomegalovirus (CMV) is a member of the β-herpesvirus subfamily, which infects 50% to 60% of the European population. Primary infections are mostly unnoticed. However, the virus reactivates from latent infections on immunosuppression, leading to graft rejection and severe pathology, such as pneumonitis in bone marrow transplantation, colitis, and retinitis in AIDS [15]. CD4^pos ^and CD8^pos ^T-cell responses against CMV have been studied by using peptides, recombinant proteins, or lysates of infected cells [16-19]. Frequencies of CMV-specific CD4^pos ^and CD8^pos ^T-cells have been shown to be extremely high in immunocompetent persons [17], and to be maintained throughout life [20]. Contributions of CD4^pos ^and CD8^pos ^T cells have been demonstrated both *in vitro *[21,22] and *in vivo*, [23,24].

Although CD4^pos ^T cells possess their own capacity to inhibit CMV replication [21,22,25], they also contribute to the differentiation and maintenance of CMV-specific CD8^pos ^T cells [23]. Moreover, anti-CMV specific effectors are increased in CD28^neg ^CD4^pos ^T cells [17,26], a population that is expanded in RA, because of TNF-α [27,28]. TNF-α has been demonstrated not only to play a prominent role in RA but also to diminish the intensity of the T-cell response [29]. Moreover, anergy of T cells was observed in RA patients [30]. Therefore, the outcome of the anti-CMV CD4^pos ^T-cell response in RA patients treated with anti-TNF-α is of interest.

Case reports have mentioned the reactivation of CMV in anti-TNF-treated patients [3]. It is thus important to know more precisely the persistence of anti-CMV memory CD4^pos ^T cells in RA. The high proportion of CMV-seropositive individuals and the high frequencies of CMV-specific T cells allow the follow-up of the Ag response *ex vivo *[17,18]. We thus have chosen to test the anti-CMV CD4^pos ^T-cell response as a model for the study of the antiviral response in RA patients whose TNF-α is neutralized with anti-TNF. We previously showed that TNF-α participates in the control of infection [25]. Because neutralization of TNF-α may alter the control of CMV, we thus measured the CD4^pos ^T-cell response in RA patients treated with anti-TNF.

Because IFN-γ produced by CD4^pos ^T cells is important in the control of CMV *in vitro *[25] and *in vivo *[21], we tested the intracellular production of IFN-γ in CD4^pos ^T cells in response to total CMV Ags in patients with RA, before and after initiation of treatment with an anti-TNF-α agent. Specific proliferation in response to CMV Ags also was investigated. Our data show that anti-TNF treatments do not impair the CD4^pos ^anti-CMV response and suggest that CMV infection remains controlled in treated RA patients latently infected with CMV.

## Materials and methods

### Patients

Patients were included in this study according to several criteria: RA diagnosed according to the 1987 ACR criteria, with active disease, eligible for a first anti-TNF therapy after failure of at least one previous disease-modifying antirheumatic drug (DMARD). Active disease was defined on a Disease Activity Score, assessed by using 28-joint counts (DAS28), above 3.2 [31]. Other associated treatments (DMARD, steroids, NSAIDs) had to be stable for 6 months before inclusion and remain stable during the study.

Patients were tested for CMV serologic status at the time of inclusion. Blood samples were drawn from CMV-seropositive patients before the beginning of anti-TNF-α treatment (day 0) and at weeks 6 and 12 after the beginning of treatment. Blood samples were drawn from CMV-seronegative patients, only once (on day 0) to evaluate the background response of unimmunized subjects.

Patients were receiving either TNF-α soluble receptor (Etanercept) or antibodies (either Infliximab or Adalimumab), according to physicians' choice. Disease Activity Score (DAS 28) was calculated at every visit, and patients' response to treatment was evaluated at week 12 according to EULAR response criteria [32].

The present study was performed with approval of the local ethics committee (CPP Toulouse II), and informed consent was obtained from all participants.

### Methods

#### Separation and preservation of cells

Blood was collected in citrate tubes, and peripheral blood mononuclear cells (PBMCs) were isolated with Lymphoprep gradient separation (Abcys Biology, Paris, France). Cells were then resuspended in 10% DMSO-40% SVF-containing medium and kept frozen in liquid nitrogen.

#### Antibodies and reagents (preparation of CMV Ag)

Anti-CD4-PE-Cy5 was purchased from eBiosciences (CliniSciences, Montrouge, France), and anti-CD28-PE and anti-IFN-γ-FITC were purchased from PharMingen (BD PharMingen, Le Pont de Claix, France). Brefeldin A was purchased from Sigma (Sigma-Aldrich, Saint-Quentin Fallavier France).

CMV total Ags were prepared as described in [16]. In brief, MRC5 cells were infected with the Towne strain of CMV at the MOI of 0.1. Cells were harvested 6 days later, washed 3 times in PBS, and lyzed by sonication. The sonicate was centrifuged, and the pellet resuspended in PBS, aliquoted, and stored at -80°C. Control Ag was prepared in parallel by using the same protocol, except that cells were uninfected.

#### Flow cytometry and intracellular IFN-γ assay

Flow cytometry for the detection of intracellular IFN-γ was performed as described by Vaz-Santiago *et al*. [16]. In brief, cells (2 × 10^6 ^in 200 μl RPMI medium, 10% SVF) were incubated with the appropriate amount of CMV Ags or control Ags for 4 h. Then Brefeldin A (4 mg/ml) was added for 12 h in 1.6 ml, and cells were left at 37°C under a humidified 5% CO_2 _atmosphere. Cells were then washed and stained for surface markers (CD28 and CD4), and then permeabilized by using the Becton Dickinson intracellular cytokine kit. Cells were then stained for intracellular IFN-γ.

#### Proliferation assay

PBMCs (2 × 10^5^) were incubated in 96-well (200 μl) U-bottomed plates in RPMI-HS (AB CMV seronegative serum) in triplicate, in the presence of either CMV Ags or control Ags. On day 5, cultures were pulsed overnight with [^3^H]thymidine ([^3^H]TdR; Amersham) (1 μCi/well). The [^3^H]TdR incorporation was determined in a beta counter and expressed as the mean of triplicates. The Stimulation Index was calculated as the ratio of means obtained by using CMV Ags over those obtained by using control Ags.

#### Statistics

After the Shapiro-Francia normality test was applied, the data were analyzed by using nonparametric tests: the Mann-Whitney two-sample statistic, the Kruskal-Wallis (several-sample statistic), and the Wilcoxon matched-pairs signed-ranks test.

Statistical analyses were performed by using Stata Statistical Software (Intercooled Stata 8.2; Stata Corporation, College Station, TX, USA).

## Results

### Characteristics of patients

Twenty-five patients (23 women and two men) median (extremes) age 55 years (31 to 81 years) years; disease duration, 12 years (2 to 26 years) were included in the present study (Table 1). CMV serologic status was positive in 17 patients but negative in eight patients. All seropositive patients had IgG but no IgM specific for CMV. In CMV-positive patients, the anti-TNF agent was added to methotrexate in nine, and to leflunomide in one, whereas it was prescribed as monotherapy in seven patients. At week 12, 11 (64.7%) patients were considered good or moderate responders (R), whereas 6 (35.3%) were considered nonresponders (NRs), according to EULAR response criteria. The overall clinical response (64.7%) observed after a 12-week period is in agreement with that reported in other studies [33].

**Table 1:** Characteristics of patients

Patients	RA (years)	Anti-TNF	Steroids	DMARDS	CMV serology status	Clinical response
1	26	ADA	PRED 5	None	+	NR
2	2	ETA	PRED 20	MTX	+	R
3	12	ETA	PRED 5	MTX	+	NR
4	17	INF	None	None	+	R
5	5	ETA	PRED 20	None	+	R
6	4	ETA	PRED 10	None	+	R
7	23	ETA	None	MTX	+	R
8	7	INF	PRED 10	None	+	R
9	26	INF	PRED 7	None	+	R
10	20	ETA	PRED 5	MTX	+	NR
11	14	INF	PRED 10	MTX	+	R
12	20	ADA	PRED 10	MTX	+	R
13	3	ADA	None	Leflunomide	+	R
14	13	INF	PRED 10	None	+	R
15	2	ETA	None	MTX	+	NR
16	12	INF	None	MTX	+	NR
17	12	INF	PRED 10	MTX	+	NR
						
18	8	INF	None	MTX	-	R
19	17	ETA	PRED 20	MTX	-	R
20	4	INF	None	AlloChrysine	-	R
21	11	ETA	PRED 5	MTX	-	NR
22	15	ETA	None	Leflunomide	-	NR
23	9	ETA	None	MTX	-	R
24	20	ADA	PRED 10	None	-	R
25	13	ADA	None	MTX	-	R

ADA, adalimumab; DMARDs, disease-modifying antirheumatic drugs; ETA, etanercept; INF, infliximab; PRED, prednisone; MTX, methotrexate; NR, nonresponder; R, responder.

### Intracellular IFN-γ response in CD4^pos ^from RA patients treated with anti-TNF

**Figure 1:**
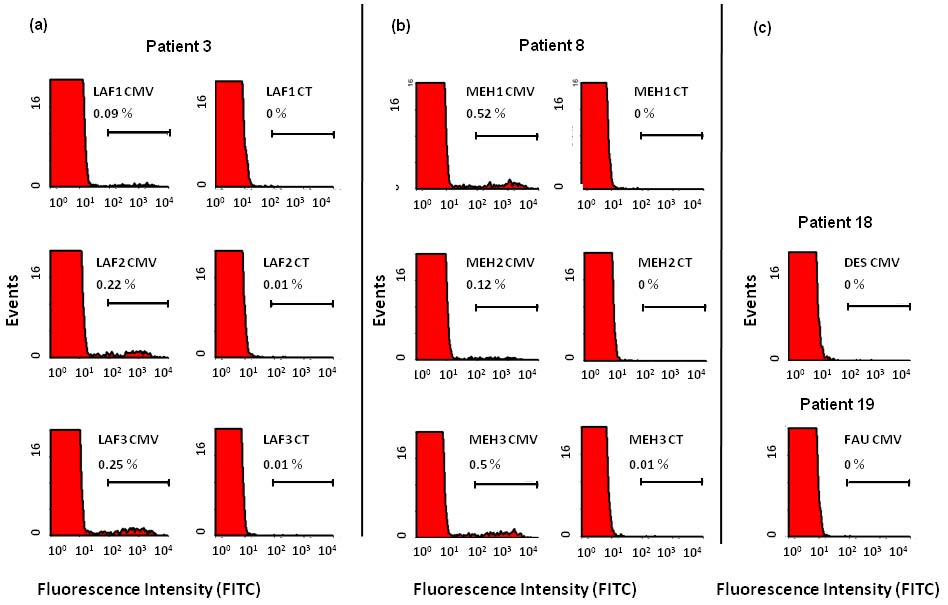
**Representative flow-cytometry profiles of the intracellular interferon (IFN)-γ response in CD4^pos ^T cells from patients receiving anti-TNF treatments**. Peripheral blood mononuclear cells (PBMCs) from two cytomegalovirus (CMV)-seropositive patients **(a, b) **were incubated with CMV Ags (CMV) and control Ags (CT), and intracellular IFN-γ production was measured with flow cytometry, as indicated in Materials and Methods. Responses from CMV-seronegative patients **(c) **are shown as controls. CD4^pos ^T-cell responses are represented as the percentage of IFN-γ^pos ^cells within the CD4^pos ^population.

The intracellular CD4^pos ^response was assessed in both CMV-seropositive and -seronegative patients. Figure 1a,b shows the percentage of CD4^pos ^T cells expressing IFN-γ in response to CMV Ags when tested in two patients seropositive for CMV in three consecutive samples (onset of anti-TNF treatment, week 6, and week 12). As expected, the response for CMV Ags was undetectable in seronegative patients (Figure 1c).

**Figure 2:**
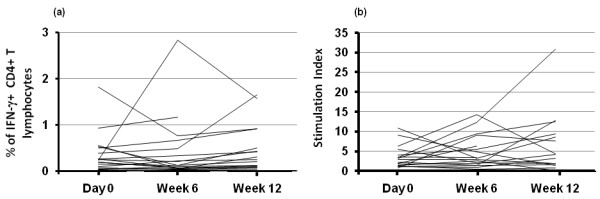
**CD4^pos ^T-cell response to cytomegalovirus (CMV) Ags in patients receiving anti-tumor necrosis factor (TNF) treatments**. **(a) **Peripheral blood mononuclear cells (PBMCs) from CMV-seropositive patients were incubated with CMV Ags, and intracellular interferon (IFN)-γ production was measured with flow cytometry, as indicated in Materials and Methods. CD4^pos ^T-cell responses are represented as the percentage of IFN-γ^pos ^cells within the CD4^pos ^population. **(b) **PBMCs were cultured for 5 days in the presence of CMV Ags, and their proliferation was evaluated by measuring the incorporation of ^3^H-thymidine. The stimulation index (S.I.) was derived by dividing the cpm obtained by using CMV-Ags by those from control Ags.

The IFN-γ response to CMV Ags (Figure 2a) was detectable in all seropositive patients and was always above that observed in the presence of control Ags (data not shown). The mean percentage of the response to CMV Ags was 0.35%, whereas the response to control Ags was less than 0.02%. No statistical significant modification of the response towards CMV Ags was noted after 6 weeks (0.43%) or 12 weeks (0.49%) of treatment with the TNF blocker. When responders and nonresponders to anti-TNF treatment were compared, no significant difference was observed at any of the time points (data not shown). The same is true when comparing the response to CMV Ags in patients treated with a monoclonal antibody or the soluble receptor of TNF.

### Proliferation response to CMV Ags

Proliferation in the presence of CMV Ags was assessed in both CMV-seropositive and -seronegative patients. As expected, seronegative patients did not respond to CMV Ags (data not shown). However, CMV-seropositive patients did respond to CMV Ags, but the response did not significantly vary over the time of exposure to anti-TNF-α (Figure 2b). Similar to what was observed with IFN-γ production, we were not able to find significant patterns in patients classified clinically as responders and nonresponders at week 12 or in patients treated with a monoclonal antibody and those treated with a soluble receptor of TNF.

### High percentages of IFN-γ-secreting cells within the CD28^neg ^CD4^pos ^population in response to CMV Ags

**Figure 3:**
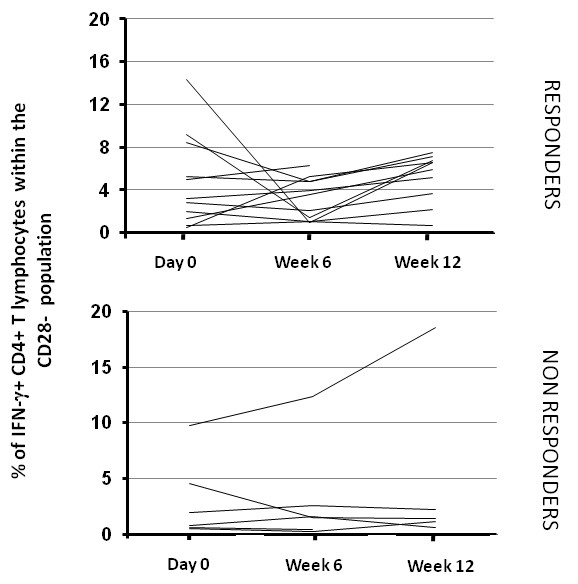
**Percentage of cytomegalovirus (CMV)-specific CD28^neg ^CD4^pos ^T cells from RA patients receiving anti-TNF treatments**. Peripheral blood mononuclear cells (PBMCs) from CMV-seropositive patients were incubated with CMV Ags, and intracellular interferon (IFN)-γ production and CD28 expression were measured with flow cytometry, as indicated in Materials and Methods. Percentages of IFN-γ-producing CD28^neg ^CD4^pos ^T cells are represented according to the responder/nonresponder clinical status of patients.

It has been reported that the CD28^neg ^population is enriched in RA patients [29] and that this phenotype is due to TNF-α [28,34]. We thus evaluated whether the clinical status of patients (responder versus nonresponder) would relate to the CD4^pos ^T-cell immunity examined through the CMV-specific CD4^pos ^population. Because the anti-CMV CD4^pos ^T-cell response is enriched in the CD28^neg ^compartment [17,26], we measured the percentage of IFN-γ^pos ^CD4^pos ^T cells within the CD28^neg ^compartment after exposure to CMV Ags. As expected, Figure 3 shows that the CD4^pos ^T cells specific for IFN-γ^pos ^were greatly enriched in the CD28^neg ^population (4.7% versus 0.35% in the total CD4^pos ^population), but, again, no significant statistical difference was observed between time points and between responders and nonresponders.

**Figure 4:**
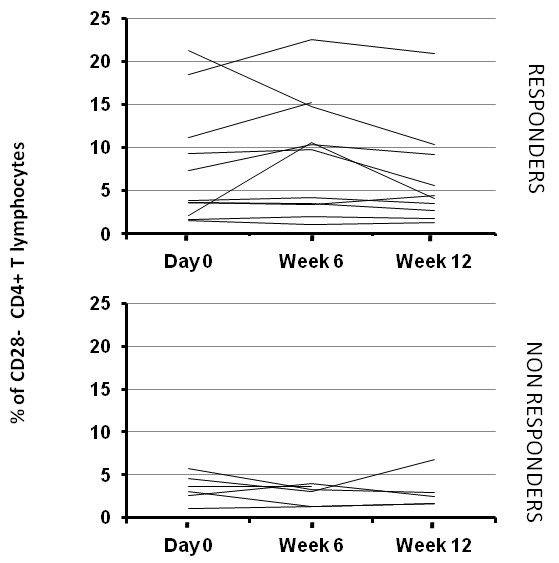
**Percentage of total CD28^neg ^CD4^pos ^T cells from anti-TNF-treated RA patients over time in patients receiving anti-tumor necrosis factor (TNF) treatments**. Peripheral blood mononuclear cells (PBMCs) from RA patients were stained for CD28 and CD4, examined with flow cytometry, and the percentages were represented according to the responder/nonresponder clinical status of patients.

We next evaluated the percentage of total CD28^neg ^among the CD4^pos ^T-cell population during anti-TNF-α treatments (Figure 4). The percentage of CD28^neg ^CD4^pos ^cells in RA patients was similar to that observed previously by Schmidt *et al*. [27,29]. As previously reported [35], this percentage did not increase over the course of the treatment (6.1%, 6.5%, and 5.2%, respectively, at day 0, week 6, and week 12) (Figure 4). However, contrary to what has been reported [35], no increase of the CD28 intensity was observed in the responders group (data not shown). Although the percentages were higher in the responders population, this difference was not statistically significant at any time point: *P *= 0.2278 at day 0; *P *= 0.056 at week 6; *P *= 0.1775 at week 12.

## Discussion

In this work, we analyzed the CD4^pos ^T-cell response to CMV total Ags to evaluate the consequences of treatments with anti-TNF agents on the viral immune memory response. We observed that the CD4^pos ^T-cell response toward CMV Ags was not altered by anti-TNF antagonists, whether soluble receptor or antibodies.

Production of IFN-γ is a marker and potent effecter of the antiviral response, especially against CMV [21,25]. In this work, total CMV Ags were used to monitor the global CD4^pos ^immune response through the IFN-γ production. It appeared that the immunity to viral CMV Ags was conserved during the course of the treatment. Primary infections by CMV are accompanied by the appearance of a high response and frequency of specific CD4^pos ^T cells, which are maintained durably [18,21,36]. The absence of modification of the anti-CMV CD4^pos ^T-cell response in most patients and slight variations in some others observed in our present study are similar to those in previous studies in normal blood donors regarding proliferation [18] and cytokine production by flow cytometry in transplant recipients [37] and HIV patients [36]. Contrary to the anti-mycobacteria CD8^pos ^T-cell response [9], the frequency of anti-CMV CD4pos T cells was not impaired by anti-TNF treatments. Our data suggest that the anti-CMV response is sufficient to control the latent CMV infection during the course of the anti-TNF treatments.

As observed in previous reports [17,26], the percentages of CMV-specific CD4^pos ^T cells in the CD28^neg ^population were high, as compared with those observed in the general CD4^pos ^T cell population. We observed that those percentages, obtained by using infected cell lysates, were lower than those observed when using synthetic peptides [17]. However, they were within the range of and in accordance with those reported with whole-cell lysate [17]. Thus, all peptides may not be available as epitopes through processing of total CMV Ags by APC.

The high proportion of CMV-specific CD4^pos ^T cells in the CD28^neg ^population was reported earlier [17,26], but had not been studied in RA. The percentage of CD28^neg ^CD4^pos ^T cells observed in the present study was similar to that observed by Schmidt *et al*. [27] in RA patients.

However, the scope of this present study was not to compare the percentage of CD28^neg ^CD4^pos ^T cells in the normal population and RA patients. We followed the proportion of CD28^neg ^CD4^pos ^T cells during the course of anti-TNF treatment. Despite the neutralization of TNF-α, the percentage of CD28^neg ^CD4^pos ^cells did not vary during the course of anti-TNF treatment in our study. The role of CD28^neg ^CD4^pos ^cells in RA is not elucidated, but it has been suggested that they do not play an aggressive role in autoimmunity and may not play a specific role in RA [34].

Hyporesponsiveness was reported in RA patients in T cells from synovial fluid [30]. The significant response of CD4^pos ^T cells from peripheral blood at day 0 of treatment and the relatively stable anti-CMV response over the course of treatment in our current study suggest (a) that anergy to anti-CMV Ags, if any, was not a prominent feature of RA patients; and (b) that anti-TNF treatments did not restore function from CD4^pos ^T cells putatively engaged in anergy or in TNF-α-induced hyporesponsiveness [29].

Several studies have analyzed the *in vitro *responses to pathogens that are at risk in RA patients treated with anti-TNF. The *ex vivo *anti-mycobacteria IFN-γ response was found to be impaired by infliximab and adalimumab [8] and was in accordance with the risk of reactivation of tuberculosis, especially with antibodies [38]. The situation regarding the antiviral immunity may be more complex and has been less explored. Although HBV has been described to reactivate on anti-TNF treatment [3], long-term safety of TNF blockers requires longer follow-up regarding HCV [3,39].

Regarding EBV, a member of the herpes family, the risk of lymphoma has been debated [3]. The anti-EBV response was found to be maintained [13], suggesting that no short-term (3 month) defect in EBV-immune surveillance occurs in patients receiving MTX or anti-TNF drugs.

A risk of varicella-zoster virus infection, another member of the herpes virus family, may be present, as reported by Strangfeld *et al*. [14]. However, *in vitro *studies of the CD4^pos ^T-cell response against varicella-zoster Ags have not been performed. Several cases of CMV infection have been reported during the course of anti-TNF treatments [3,40,41]. However, because patients received concomitant immunosuppressive treatments, it is difficult to establish a link between anti-TNF treatment and reactivation of CMV.

Our present data argue in favor of the maintenance of anti-CMV immunity during anti-TNF treatments. This is of importance in light of previous observations that TNF-α is an important component of the anti-CMV control *in vitro *[25,42]. Our current study and former published reports [8,13] suggest that *in vitro *responses to viral proteins or peptides are of help to identify risks of viral infection in patients treated with anti-TNF. In addition, the conservation of anti-CMV CD4^pos ^T cell immunity during anti-TNF treatment suggests that vaccinations can be envisaged during treatment by anti-TNF. However, until safety data are available, live attenuated virus vaccines should be contraindicated in RA patients.

## Conclusions

We have used the anti-CMV CD4^pos ^T-cell response as a test for the integrity of the antiviral immune response during anti-TNF treatments. Our data show that the anti-CMV CD4^pos ^T-cell IFN-γ and proliferative responses are maintained during anti-TNF treatments. No modification of the percentage of specific or total CD28^neg ^CD4^pos ^T cells during anti-TNF treatments was observed. Because CD4^pos ^T cells are an important component of the anti-CMV immunity, our observations suggest that CMV infections are well controlled during anti-TNF treatments and bring new insight into the current knowledge of the risks of infection in patients treated with anti-TNF-α biotherapies.

## Abbreviations

Ags: antigens; CMV: cytomegalovirus; RA: rheumatoid arthritis.

## Competing interests

The authors declare that they have no competing interests.

## Authors' contributions

J-LD, ACo, and ACa designed the study. DN participated in the design of the study and performed the statistical analysis. J-LD and J-FB performed the experiments. J-FB, BJ, ACo, and ACa enrolled patients. J-LD, ACo, and ACa wrote the manuscript. All authors helped to draft the manuscript. All authors read and approved the final manuscript.
